# Bilateral diffuse retinal pigment epithelium proliferation induced by choroidal inflammation: A case report

**DOI:** 10.1097/MD.0000000000018152

**Published:** 2019-11-22

**Authors:** Miyuki Tanaka, Koju Kamoi, Natsuko Nagaoka, Tomoka Ishida, Hisako Karube, Hiroshi Takase, Kyoko Ohno-Matsui

**Affiliations:** a Department of Ophthalmology and Visual Science, Graduate school of Medical and Dental Sciences, Tokyo Medical and Dental University; b Department of Ophthalmology, Musashino Red Cross Hospital, Tokyo, Japan.

**Keywords:** bilateral diffuse uveal melanocytic proliferation, choroidal inflammation, ocular inflammation, retinal pigment epithelium proliferation, vogt-koyanagi-harada disease

## Abstract

**Rationale:**

Proliferation of retinal pigment epithelium (RPE) is typically observed in limited ocular disorders, in connection with the local mechanism of RPE proliferation-mediated wound repair. Bilateral and diffuse type RPE proliferation is considered to be associated with paraneoplastic syndromes, such as a bilateral diffuse uveal melanocytic proliferation. However, other reported diseases that induce bilateral diffuse RPE proliferation are quite rare, especially for patients who are considered to have a non-malignant status.

**Patient concerns:**

The bilateral eyes of a 47-year-old woman with bilateral ocular inflammation, presented united multiple small to medium white retinal lesions during the disease progress.

**Diagnoses:**

Optical coherence tomography showed scattered serous retinal detachments, choroidal folds, choroidal thickening and diffuse RPE proliferation. As autofluorescence and angiography showed a “giraffe pattern”, bilateral diffuse uveal melanocytic proliferation was suspected. However, systemic investigations identified no malignancy. In consideration of the above findings, choroidal inflammation was thought to be the major cause of this condition.

**Interventions:**

The patient was administered intensive systemic steroids. Over the next 2 months, the amount of steroid was tapered off.

**Outcomes:**

After administration, the bilateral diffuse RPE proliferation settled down. During the 2-year follow-up, there was no recurrence of ocular inflammation and diffuse RPE proliferation, or any other malignancy found.

**Lessons:**

This finding demonstrates that bilateral diffuse RPE proliferation can be generated as a secondary phenomenon of choroidal inflammation in patients with a non-malignant status.

## 1 Introduction

Proliferation of retinal pigment epithelium (RPE) is typically observed in limited ocular disorders, such as proliferative (diabetes) retinopathy, age-related macular degeneration and retinal detachment, in connection with the local mechanism of RPE proliferation-mediated wound repair.^[1,2]^ However, not only is the incidence of bilateral and diffuse type RPE proliferation quite low, the mechanism responsible for the disease is not well understood.

One of the typical diseases that has been reported to cause bilateral diffuse proliferation of RPE is bilateral diffuse uveal melanocytic proliferation (BDUMP).^[3]^ BDUMP is a form of a paraneoplastic syndrome that causes proliferation of the melanocytes bilaterally, which causes visual impairment due to the rapidly progressing exudative retinal detachment and cataract formation.^[4]^ Although it has been reported that the proliferation of melanocytes in the choroid and RPE is considered to be caused by humoral factors produced from tumor,^[5]^ the details of the pathogenesis, pathophysiology and treatment method are still unclear. Other reported diseases that induce bilateral diffuse RPE proliferation are quite rare, especially for patients who are considered to have a non-malignant status.

Here, we report bilateral diffuse RPE proliferation, which was a secondary phenomenon caused by an ocular inflammation that was predominantly found to be choroidal inflammation, in a patient considered to have a non-malignant status.

## 2 Case report

**Figure 1:**
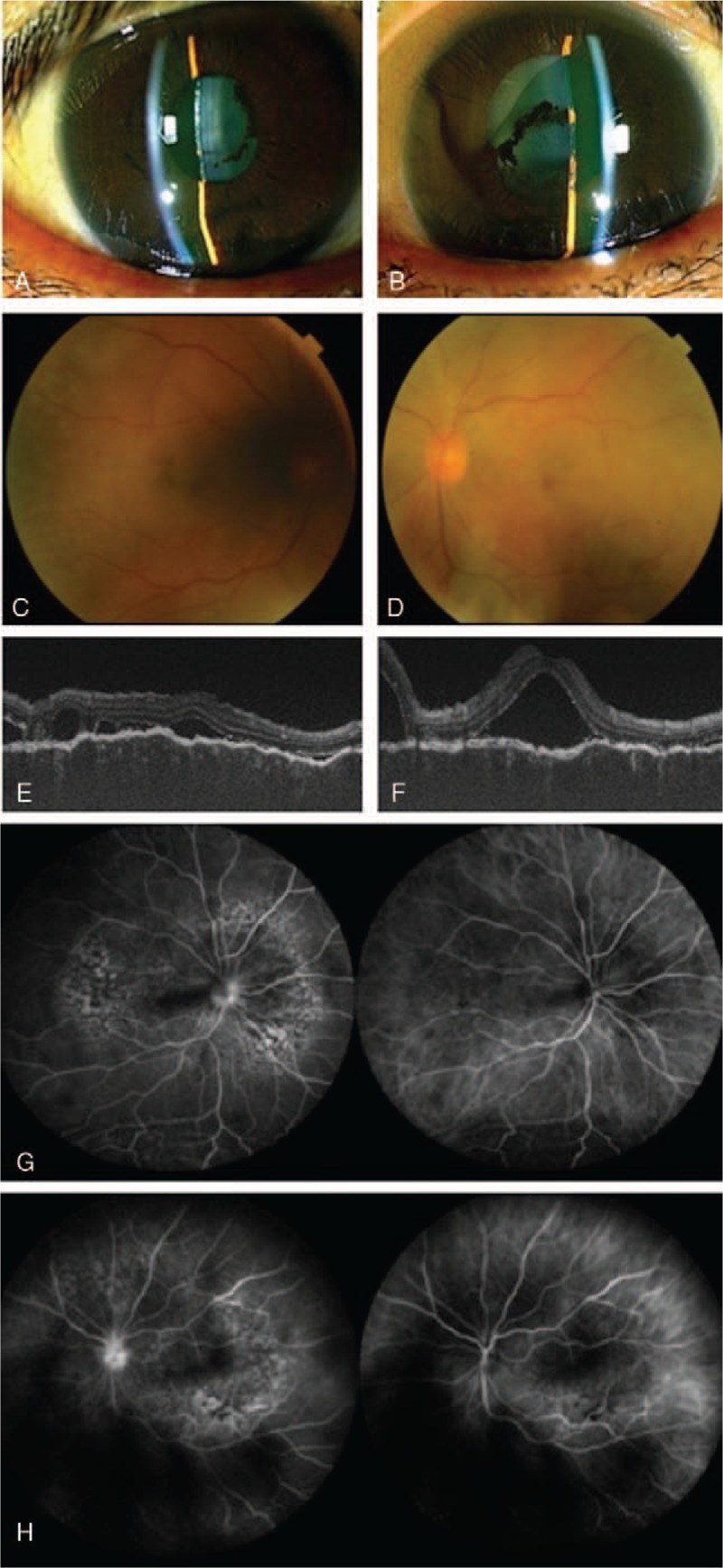
Ocular findings at the first visit: Anterior inflammation and accompanying posterior synechia of the iris were seen in the anterior segment (A, B). Multiple small to medium circular white lesions were seen in the retina (C, D). OCT detected choroidal folds, choroidal thickening and exudative serous retinal detachment. FA (G, H; left side) demonstrated the presence of hyperfluorescence of the optic nerve head and granular hyperfluorescence near the arcade blood vessels. IA (G, H; right side) revealed a filling delay of the choroidal vessels and poor visualization of the vessels (A, C, E, G: right eye. B, D, F, H: left eye).

A 47-year-old woman visited an ophthalmology clinic in January 2017 with foggy vision and vision loss. Although her medical history included uveitis 15 years prior, she had no other ocular disease, systemic disease such as inflammatory diseases, or malignant disorders. At the first visit, the best corrected visual acuity (BCVA) was 20/30 and 20/40 in her right and left eye, respectively, while the intraocular pressure was 13 and 11 mmHg in her right and left eye, respectively. In the anterior segment findings, no keratic precipitate, 1+ cells and 1+ flare in the anterior chamber, and remarkable posterior synechia were detected in both eyes (Fig. 1A and B). Fundus examinations revealed mild redness and swelling of the optic disk, with multiple small to medium white retinal lesions (partly united) seen at the retina of both eyes. (Fig. 1C and D). Optical coherence tomography (OCT) identified scattered serous retinal detachments, choroidal folds and diffuse choroidal thickening in both eyes (Fig. 1E and F). Although fluorescein angiography (FA) showed hyperfluorescence at the optic disc and granular hyperfluorescence around the arcade vessels (Fig. 1G, H, left), leakage and pooling from multiple pinpoints, which is a typical feature in Vogt-Koyanagi-Harada disease (VKH), were undetectable. Indocyanine green angiography (IA) identified filling delay of the choroidal vessels and poor visualization of the vessels (Fig. 1G, H, right). Systemic investigation, blood and urine laboratory tests revealed no abnormal findings. Cerebrospinal fluid examination showed no evidence of aseptic meningitis, while auditory examination revealed no sensorineural deafness. Therefore, a VKH diagnosis could not be determined based on the ocular findings and systemic investigations.

**Figure 2:**
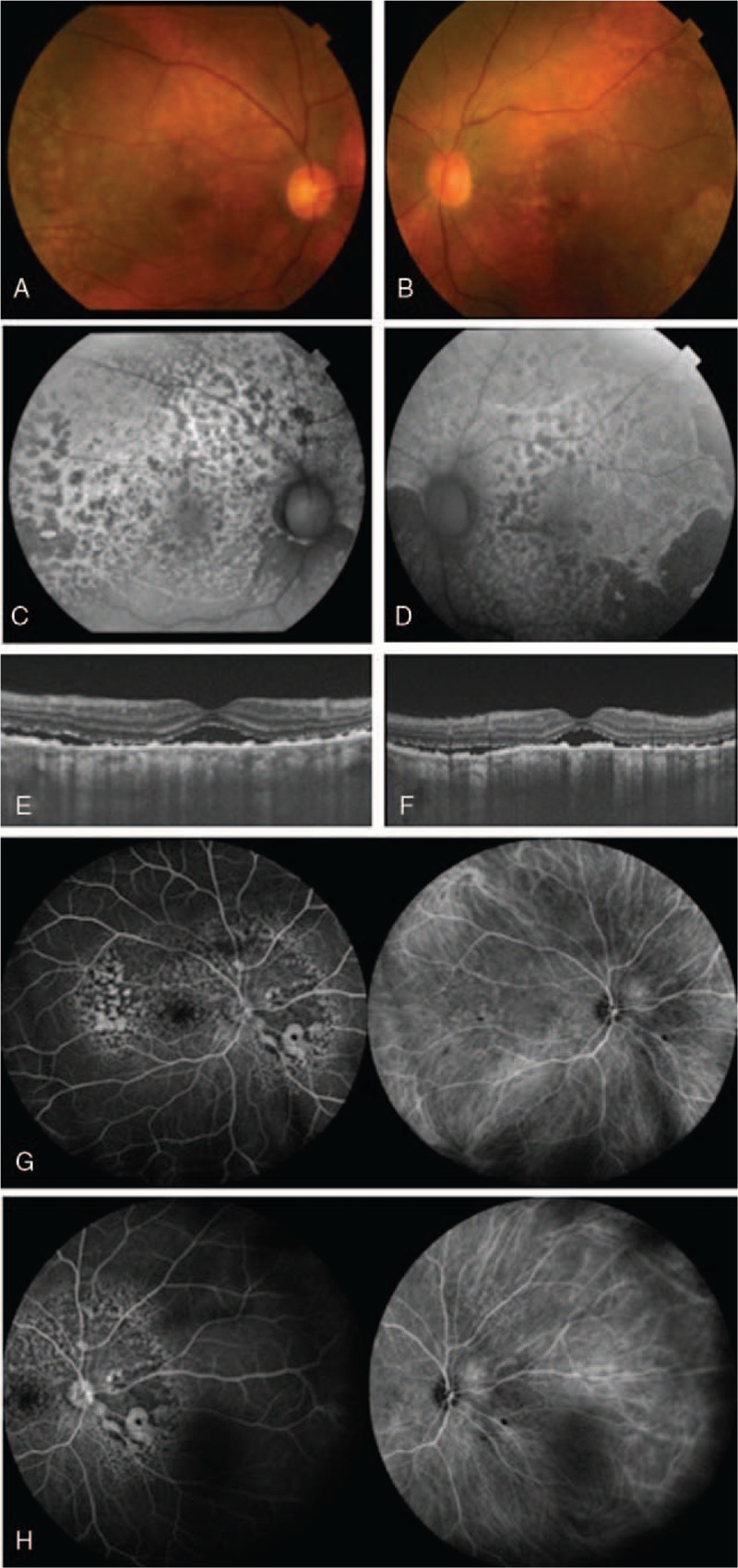
Ocular findings two weeks after onset. Multiple coalescing circular depigmented spots and pigmentation were seen (A, B). Multiple nummular hyperfluorescent lesions surrounded by zones of hypofluorescence (giraffe pattern) were detected by FAF (C, D). OCT showed exudative serous retinal detachment and the new appearance of hyperplasia of the retinal pigment epithelial (E, F). FA showed the presence of multiple nummular hyperfluorescent lesions surrounded by zones of hypofluorescence (giraffe pattern) (G, H, right). IA demonstrated the presence of the giraffe pattern hypercyanescent spots (G, H, left). The patterns seen by the FA and IA evaluations were the inverse of the pattern found for the FAF (right eye: A, C, E, G. left eye: B, D, F, H).

In consideration of these findings, it was presumed that choroidal inflammation strongly contributed to the pathology of this case. As a result, we decided to perform steroid treatment in the patient. Steroid pulse (1000 mg / day for 3 days) followed by an oral administration (starting at 50 mg / day) led to improvement in the choroidal folds and choroidal thickening. However, the serous retinal detachment tended to remain. Two weeks after the onset of the disease, bilateral diffuse RPE proliferation was clearly observed. A fundus photo showed multiple, coalescing, circular depigmented plaques and pigmentation, which were considered to be caused by hyperplasia of the RPE (Fig. 2A and B). In addition, multiple nummular hyperfluorescent lesions surrounded by zones of hypofluorescence, which are referred to as the ‘giraffe pattern’, was observed by fundus autofluorescence (FAF) (Fig. 2C and D), and OCT revealed hyperplasia of the RPE (Fig. 2E and F). Hyperplasia of the RPE was seen not only at the detached area of the retina, but also at the attached area of the retina. Multiple nummular hyperfluorescent lesions surrounded by zones of hypofluorescence (giraffe pattern) were seen by FA (Fig. 2G, H, right). Giraffe pattern hypercyanescent spots were also shown by IA (Fig. 2G, H, left). The giraffe patterns observed when using FA and IA were the inverse of the pattern seen with FAF.

Based on these findings, we considered BDUMP to be the most probable differential diagnosis of the disease that was associated with the hyperplasia of the RPE. As a result, we performed whole-body investigations using computed tomography, magnetic resonance imaging, positron emission tomography, and echo in order to try and detect the presence of malignant tumors in this patient. However, findings of these evaluations did not suggest the presence of any malignant tumors.

**Figure 3:**
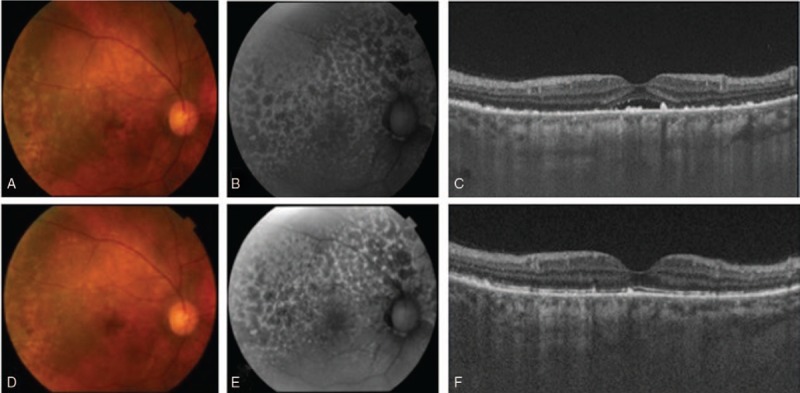
Ocular findings at 2 (A, B, C) and 4 (D, E, F) months after onset in the right eye. There was a tendency for the depigmented lesion to disappear (A, D). FAF showed that the giraffe pattern still remained (B, E). OCT showed that there was a gradual disappearance of the serous retinal detachment and a gradually reduction in the diffuse RPE proliferation (E, F).

During the 2-month steroid treatment, there was improvement of the choroidal folds, choroidal thickening and serous retinal detachment observed in the patient (Fig. 3A–C). Corresponding to this improvement of the choroidal inflammation, we also observed a reduction of the hyperplasia of the RPE after the 4-month treatment (Fig. 3D–F). Although there was a tendency for the depigmented lesion to disappear, the giraffe pattern remained visible during FAF during these periods. BCVA also improved to 20/13 and 20/20 in the right and left eye, respectively. In addition, during the next 2-year follow-up period, there was no recurrence of the choroidal inflammation and bilateral diffuse RPE proliferation. The BCVA had remained good, and there has been no further appearance of malignant tumors.

## 3 Discussion

BDUMP is a representative disease that is associated with bilateral and diffuse proliferation of the RPE. The clinical features of this disease include:

1. multiple circular or oval orange patches (decolorizing spots) at the level of the RPE,
2. multifocal early hyperfluorescence corresponding patches,
3. extensive thickening of the uvea with focal nodules,
4. exudative retinal detachment, and
5. rapid cataract progression.

In addition, other typical findings include the recent OCT images of the BDUMP identified hyperplastic images of the RPE and the giraffe pattern observed by angiography and autofluorescence.^[3]^

When this case was compared to the typical findings for BDUMP, the common ophthalmological findings included bilateral diffuse hyperplasia of the RPE and serous retinal detachment. Therefore, we listed BDUMP as a differential diagnosis in this case. However, extensive thickening of the uvea with focal nodules and rapid progression of cataract, which are typical features of BDUMP, were not observed in this case. In addition, since BDUMP is considered to be a paraneoplastic syndrome and is often observed prior to a malignant tumor,^[6]^ a systemic investigation was repeated in this case for 2 years from the initial onset. However, there was no malignant tumor ever observed. Thus, the proliferation of RPE in this case was considered to differ from the pathological mechanism of BDUMP.

In this case, we strongly suspected that inflammation played an important role in the pathogenesis of the proliferation of RPE, as OCT showed that the anterior segment inflammation was clearly observed and there was choroidal folds/choroidal thickening. Furthermore, IA showed that there was a filling delay of the choroidal large blood vessel, thereby indicating choroidal inflammation. Although VKH was considered as a differential diagnosis in this case, diffuse proliferation of the RPE and the presentation of the giraffe pattern in this case are not commonly seen in VKH. Moreover, the specific systemic features of VKH, such as aseptic meningitis and sensorineural hearing loss, were not seen in this patient. Therefore, VKH was excluded as a differential diagnosis. It has been reported in previous studies that pseudotumoral retinal pigment epithelial proliferation occurred with VKH.^[7]^ The specific findings in these cases were the formations of local nevus-like elevated lesions, which suggested the proliferation of choroidal melanocytes. On the other hand, our current case did not exhibit any elevated lesions.

From the aspect of angiography, other differential diagnoses including idiopathic uveal effusion and blood diseases such as lymphoma/leukemia, which cause “giraffe pattern” changes,^[8]^ were all ruled out after ophthalmologic and systemic examinations. In addition, local proliferation of RPE induced diseases such as proliferative (diabetes) retinopathy, age-related macular degeneration, and retinal detachment were also ruled out based on the fundus findings.

From a treatment perspective, it has been shown to be difficult to improve the vision in BDUMP patients when just using steroid therapy.^[9]^ However, in our current case, steroid treatment was clearly effective on the choroidal inflammation, with a subsequent reduction of the hyperplasia of the RPE observed after the treatment. This strongly supported the theory that bilateral diffuse RPE proliferation was associated with the choroidal inflammation.

In conclusion, the findings described here support the hypothesis that diffuse RPE proliferation can be induced by ocular inflammation, even in patients without any malignant tumors. Furthermore, choroidal inflammation played a major role in generating bilateral diffuse RPE proliferation.

## Author contributions

**Conceptualization:** Koju Kamoi.

**Data curation:** Miyuki Tanaka, Natsuko Nagaoka, Tomoka Ishida, Hiroshi Takase.

**Formal analysis:** Miyuki Tanaka, Koju Kamoi.

**Investigation:** Miyuki Tanaka, Koju Kamoi.

**Supervision:** Koju Kamoi, Kyoko Ohno-Matsui.

**Writing – original draft:** Miyuki Tanaka, Koju Kamoi.

**Writing – review & editing:** Miyuki Tanaka, Koju Kamoi, Natsuko Nagaoka, Tomoka Ishida, Hisako Karube, Hiroshi Takase, Kyoko Ohno-Matsui.
